# Testing as an approach to control the Corona epidemic dynamics and avoid lockdowns

**DOI:** 10.1007/s10368-021-00495-5

**Published:** 2021-02-19

**Authors:** Thomas Gries, Paul J. J. Welfens

**Affiliations:** 1 Center for International Economics at Paderborn University, Chair for International Growth and Business Cycle Theory, Warburger Str. 100, 33098 Paderborn, Germany; 2 European Institute for International Economic Relations (EIIW) at the University of Wuppertal, Schumpeter School of Business & Economics, Rainer-Gruenter-Str. 21, D-42119 Wuppertal, Germany; 3 Chair for Macroeconomics and Jean Monnet Chair for European Economic Integration, University of Wuppertal, Wuppertal, Germany; 4 IZA, Bonn, Germany; 5 AICGS/Johns Hopkins University, Washington, DC USA

**Keywords:** Corona testing, Epidemic, Lockdowns, Cost pandemic response, World economy, H12, H51, I10, I18

## Abstract

Vaccinations, lockdowns and testing strategies are three potential elements of an effective anti-coronavirus, and in particular Covid-19, health policy. The following analysis considers - within a simple model - the potentially crucial role of a Corona testing approach in combination with a quarantine approach which is shown herein to be a substitute for broad lockdown measures. The cost of lockdowns/shutdowns are rather high so that – beyond progress in terms of a broad vaccination program – a rational testing strategy should also be carefully considered. Testing has to be organized on the basis of an adequate testing infrastructure which could largely be implemented in firms, schools, universities and public administration settings. As regards the cost of a systematic broad Covid-19 testing strategy, these could come close to 0.5% of national income if there are no vaccinations. The Testing & Quarantine approach suggested here – with tests for symptomatic as well as asymptomatic people - is based on a random sampling and would require rather broad and frequent testing; possibly one test per person every 7–10 days. At the same time, one should consider that the cost of further lockdowns/shutdowns of a duration of 1 month could be very high, such that a standard cost benefit analysis supports the testing approach suggested herein. Also, an optimal policy mix could be designed where both vaccinations and testing play a crucial role. As of late January 2021, no further lockdowns in Germany and other OECD countries would be necessary if a broad testing infrastructure can be established rather quickly. This in turn will reinforce economic optimism and help to jumpstart economic growth in Europe, the US and Asia in a solid way. The basic logic of the testing approach pointed out here for industrialized countries could also be applied in developing countries. The approach presented is complementary to the IMF analysis of Cherif/Hasanov.

## Introduction

In early 2021, vaccination programs aimed at combatting the spread of Covid-19 infections have been initiated in most OECD countries so that the Corona dynamics and the numbers of people becoming infected should begin to slow down, however, the number of doses of the appropriate vaccinations which are available are still rather modest in many countries. At the end of 2020, the situation in many countries had become rather dramatic such that new lockdowns/shutdowns had been adopted in numerous countries in late December 2020/early January 2021. Hence the vaccination programs are beginning in the context of an overall national epidemic situation which is still rather tense within the medical systems of several OECD countries. As regards Covid-19 fatality ratios and Covid-19 infections, several papers (see, for example, Bretschger et al. 2020) have looked into key empirical aspects which show that some of the main drivers of fatality ratios in OECD countries differ from those in developing countries. As regards the infection dynamics, it is also apparent that geographical aspects – partly affecting the intensity of contacts – matter (Chen et al. 2020). The IMF’s October 2020 economic outlook report has clearly indicated that the Corona World Recession has resulted in high national and global output costs in 2020 (IMF 2020).

The following Table 1 shows some basic statistics on Covid-19 fatality ratios and Covid-19 infections ratios plus the absolute total death figures and total case figures in OECD countries plus China by the end of December 31, 2020. It is rather surprising that China – with a population of approximately 1.4 billion - as the presumed starting point of the pandemic has recorded only about 5000 total deaths from the disease which is the same order of magnitude as Greece with almost 11 million inhabitants.

**Table 1:** Covid-19 Fatalities and Covid-19 Infections (Absolute) as well as Covid-19 Fatality Ratios and Covid-19 Infection Ratios in OECD countries plus China; table ranked by fatality ratio in column (1) in descending order (2020)

Rank	Country	(1)	(2)	(3)	(4)
		Total_deaths_per_mn	Total_cases_per_mn	Total_deaths	Total_cases
1	Belgium	1684.96	55,782.35	19,528	646,496
2	Slovenia	1297.30	58,757.09	2697	122,152
3	Italy	1226.54	34,851.18	74,159	2,107,166
4	Spain	1087.31	41,242.09	50,837	1,928,265
5	United Kingdom	1084.49	36,770.92	73,622	2,496,231
6	Czechia	1081.34	67,108.25	11,580	718,661
7	United States	1044.51	60,326.06	345,737	19,968,087
8	France	992.12	41,022.25	64,759	2,677,666
9	Hungary	987.23	33,385.33	9537	322,514
10	Mexico	975.76	11,060.76	125,807	1,426,094
11	Switzerland	883.34	52,260.65	7645	452,296
12	Chile	868.79	31,856.37	16,608	608,973
13	Sweden	864.12	43,307.98	8727	437,379
14	Colombia	849.26	32,285.41	43,213	1,642,775
15	Luxembourg	790.76	74,148.21	495	46,415
16	Poland	754.47	34,213.85	28,554	1,294,878
17	Austria	690.84	40,062.07	6222	360,815
18	Portugal	677.28	40,569.76	6906	413,678
19	Netherlands	672.61	47,177.59	11,525	808,382
20	Lithuania	535.58	51,639.96	1458	140,579
21	Greece	464.16	13,321.43	4838	138,850
22	Ireland	453.04	18,587.04	2237	91,779
23	Canada	414.18	15,484.25	15,632	584,409
24	Germany	403.31	21,012.62	33,791	1,760,520
25	Slovakia	391.60	32,885.48	2138	179,543
26	Israel	384.15	48,900.70	3325	423,262
27	Latvia	336.65	21,685.91	635	40,904
28	Turkey	247.58	26,187.77	20,881	2,208,652
29	Denmark	224.09	28,333.95	1298	164,116
30	Estonia	172.63	21,100.02	229	27,990
31	Finland	101.25	6516.66	561	36,107
32	Iceland	84.98	16,861.54	29	5754
33	Norway	80.42	9143.11	436	49,567
34	Australia	35.65	1114.71	909	28,425
35	Japan	26.03	1864.47	3292	235,811
36	South Korea	17.89	1204.80	917	61,769
37	New Zealand	5.18	448.34	25	2162
38	China	3.32	66.67	4782	95,963

Source: Own representation of data available from Our World In Data (OWID)

The worst performers in terms of Covid-19 fatalities in the group of OECD countries plus China were Belgium, Slovenia, Italy, Spain, the United Kingdom, Czechia, US, France, Hungary and Mexico; while the ten best performers were Denmark, Estonia, Finland, Iceland, Norway, Australia, Japan, the Republic of Korea, New Zealand and China. The fatality ratio in Belgium was 10 times as high as in Finland and four times as high as in Germany; the variance within the European Union (EU28 or EU27: EU without UK) was thus considerable in 2020.

With a relatively low incidence – the ratio of infected per 100,000 people within 1 week – health authorities are capable of tracking the contacts of those who have been tested positive. In Germany, the critical incidence was estimated at 50 by the government at the beginning of the Corona crisis in March and April 2020 (for age brackets and all weeks see Appendix), but this figure was not raised by the end of 2020: The lack of a modern, strongly digitalized health administration apparently translates into a modest tracing capacity on the part of health authorities in Germany. One should add that the situation in many EU countries is similar, with many national health authorities facing challenges similar to those seen in Germany. This is in stark contrast to Taiwan, South Korea and Japan, where digital tracing technologies have played a crucial role and have helped to achieve a relatively good performance during the course of the pandemic, namely low infection ratios and low fatality ratios when compared to Western economies.

High infection rates have clearly undermined the supply side of the economy through a negative labor input effect, but there were also psychological effects which resulted in negative demand-side macro effects which undermined economic growth and employment creation in 2020. The first – rather comprehensive - lockdowns in Italy, Spain, the UK, France and Germany plus the US brought a sharp contraction of output as is shown in Table 2.

**Table 2:** Estimated Cost of Covid-19 Lockdowns (Spring 2020)

Country	(1)	(2)	(3)	(4)	*(5)*	*(6)*	*(7)*	*(8)*
	GDP ($bn, PPP)	ΔIMF GDP forecast (%)	ΔGDP forecast ($bn, PPP)	Actual lockdown length (days)	*ΔGDP per day ($bn, PPP)*	Est. cost of lockdown per day ($bn, PPP)*	Est. cost of lockdown per day ($mn, PPP)*	Est. cost of lockdown per day (% GDP)*
DK	300	−8.4	−25.20	28	*−0.900*	*0.135*	*135*	*0.045*
NZ	200	−9.9	−19.80	35	*−0.566*	*0.085*	*84.86*	*0.042*
DE	4160	−8.2	−341.12	37	*−9.219*	*1.383*	*1382.92*	*0.033*
BE	540	−8.2	−44.28	47	*−0.942*	*0.141*	*141.32*	*0.026*
IT	2250	−9.7	−218.25	57	*−3.829*	*0.574*	*574.34*	*0.026*
UK	2980	−8.0	−238.40	49	*−4.865*	*0.730*	*729.80*	*0.024*
KR	2310	−3.4	−78.54	29	*−2.708*	*0.406*	*406.24*	*0.018*
US	20,290	−8.0	−1623.20	94	*−17.268*	*2.590*	*2590.21*	*0.013*
CN (Hubei)	1290	−4.6	−59.34	62	*−0.9571*	*0.144*	*143.56*	*0.011*

Source: Based on data from Online Appendix 9, Supplementary Material, of Balmford et al. (2020) and own calculations

Balmford et al. (2020) make the argument that the cost of a lockdown can be estimated at 0.15 of the total economic cost – the estimated cost attributed to a lockdown is thus estimated here as ΔGDP per day × 0.15 to arrive at an estimated daily cost of the lockdown in billion, million and as a percentage of GDP

As regard the pandemic, there are three main measures to control infection dynamics:

- Lockdowns of part of the population - which have the disadvantage that such measures impair production and also dampen consumption and *a fortiori* investment plus output growth and employment.
- Selective quarantine measures - which in 2020 were typically applied in the context of travelers returning from international visits/or after specific testing for the virus. Testing was also a familiar approach in care homes and hospitals in OECD countries during 2020. Quarantine can be imposed on those who are considered to represent those who stand for a high risk of spreading the infection – individuals with a positive test result are routinely sent into quarantine for several days. Effectively imposing a quarantine (e.g., people strictly confined to home) is not an easily effective tool as long as there is no electronic device, such as effective epidemic tools for tracking based on mobile phone technology, which facilitate quarantine decisions and monitoring for a limited time. To some extent, social peer group pressure could substitute for technical monitoring devices: Teachers and pupils in a given school will want not to suffer any negative reputational damage within their respective peer group and thus most of them can be expected to follow quarantine requirements as the consequence of a positive test. People working together within a certain firm could also be expected to consider social norms as a deterrent to “cheating” in the field of health policy measures known to be of public interest.
- Corona vaccinations of people above a certain age – typically above the age of 16 – represent a novel approach to controlling the pandemic; given the scarcity of vaccines in Europe and worldwide it might take well until late 2021 for most countries in the world to have achieved herd immunity.

Our particular interest puts a focus on the role of testing which requires government a) to develop a physical testing infrastructure and b) to organize and implement selective and regular testing so that the speed of the spread of the coronavirus can be controlled in an effective way. The testing proposal developed here is partly in line with the approach suggested by Cherif and Hasanov (2020) in an IMF working paper. Our analysis adds simulations and particular policy perspectives to the debate. One also may emphasize that the Testing & Quarantine (T&Q) approach developed here clearly shows that a lockdown can be fully avoided with an adequate testing regime which, however, requires government to strongly invest into a national (and possibly international) testing infrastructure. Moreover, one should emphasize that the approach presented is powerful in terms of fighting the epidemic, generating cost savings for society, stimulating the economic recovery and maintaining individual freedoms.

The following analysis considers in some basic aspects of controlling corona epidemics at the national and international level in Section 2. Section 3 presents basic modelling of testing strategies and cost considerations, while Section 4 is a discussion of key policy conclusions.

## Alternative measures to control Corona epidemic dynamics

Besides vaccinations, there are the two main anti-epidemic measures, namely lockdowns/shutdowns on one hand, and Testing & Quarantine on the other, which are expected to reduce the contact intensity between people and thus slow the spread of the virus considerably. Lockdowns restrict the mobility of all individuals considerably which implies a reduced contact intensity. By contrast, a T&Q approach will only lead to the imposition of restrictions on a rather small number of individuals and thus generates positive welfare effects. A broad T&Q approach implies that all age groups in society are tested with a certain frequency and depending on the “standard individual contact patterns in the respective age group”, a positive test for one person would imply that this person and his/her main contact persons would all go into quarantine for a certain period.

As regards options for controlling coronavirus epidemic dynamics, one may initially emphasize that lockdowns/shutdowns are fairly expensive measures intended to bring the speed of the spread of Covid-19 infections under control. The cost of the first lockdown/shutdown in spring 2020 reached about $9.2 billion (in PPP figures) per day in Germany, $4.8 billion in the UK and $17.2 billion in the US. On a per person basis, the respective figures are $58 per capita per day in Germany, $7.4 per capita per day in the UK and $11 per capita per day in the US – these findings are based on figures in Table 2. Depending on the length of the respective national or regional shutdowns, there will be a certain negative impact on expected output growth (on IMF GDP forecast changes, see Table 2 with a range of GDP impacts of −3.4% for the Republic of Korea to −9.7% in Italy and − 9.9% in New Zealand, respectively). The definitions of what constitutes a lockdown apparently differ across OECD countries (as do estimates of the associated economic cost) and conditions have certainly varied even across US states in spring 2020, but there is a broad agreement that many Western countries have indeed applied lockdown and shutdown measures (broadly defined) in the first half of 2020. According to Balmford et al. (2020), who suggest that 15% of the GDP decline in spring 2020 can be attributed as the cost of a lockdown, the estimated costs of such lockdowns per day for selected countries was in the range of 0.045% of GDP in Denmark (top figure) to 0.011% of GDP for Hubei Province in China and 0.013% of GDP for the US. It is possible that the economic costs of the second lockdown – which came in many countries in late autumn 2020 - was somewhat lower as individuals and firms had been able to adjust based on the experience of the first lockdown. The output cost of lockdowns and shutdowns can be mitigated by adequate liquidity supporting measures on the part of government – as well as by other complementary measures – as discussed by Pfeifer et al. (2020). On the costs of lockdowns, see also Gros (2020) and Layard et al. (2020).

It is remarkable that the shortest shutdown among the countries shown in the subsequent table, Table 2, is Denmark, followed by the Republic of Korea and New Zealand. As is shown in Table 3, Denmark is – disregarding the rather small country of Luxembourg – the leading OECD country when it comes to testing in the year 2020.

**Table 3:** Total tests (cumulated for 2020) for COVID-19 per 1000 inhabitants in 35 OECD countries based the latest data available by December 31, 2020

Rank	Country	Total_tests_per_thousand
1	Luxembourg	2661.80
2	Denmark	1815.27
3	Israel	933.14
4	United Kingdom	766.85
5	United States	749.20
6	Iceland	711.01
7	Lithuania	608.77
8	Belgium	603.37
9	Norway	523.23
10	Portugal	522.07
11	Ireland	490.58
12	Estonia	486.97
13	Latvia	471.51
14	Spain	467.55
15	Finland	450.33
16	Australia	448.19
17	Italy	445.34
18	Austria	428.29
19	Germany	415.37
20	Switzerland	381.49
21	Canada	372.04
22	Czechia	359.33
23	Slovenia	348.97
24	Chile	343.69
25	Netherlands	298.63
26	Turkey	295.84
27	New Zealand	293.31
28	Greece	271.06
29	Slovakia	267.02
30	Hungary	234.13
31	Poland	186.16
32	Colombia	120.61
33	South Korea	81.03
34	Japan	35.90
35	Mexico	25.37

Source: Own representation of data available from Our World In Data (OWID)

Not all countries used the same cut-off date in December (for further information, see the Appendix

As regards testing, we do not only have figures for OECD countries, but we also have specific figures on the number of persons tested (cumulative) by age groups as is show in Table 4. As regards the incidence statistics by age in Germany, it is quite apparent that the incidence figure (the number of reported infections per 100,000 per week) are much higher in the age group of 80 years of age and up, than in the age brackets below. One possible implication for policymakers could be to offer digital tracing devices for free to this elderly ag group (80+); this would make particular sense if the whole system is fully automated and results are digitally reported to a special “entrusted medical surveillance group”. This institutional construction should include the condition that the automatic digital reporting will be phased out by mid-2021 which seems to be a safe date to assume that all people in the relevant age group, i.e. the 80+ group, have been vaccinated and the threat of the spread of the coronavirus has generally reduced.

**Table 4:** Number of people tested in Germany by age group and positive results

Age group	Total	Total number of positive tests	Positive tests in %	Age group share in %
0–4	164,172	8168	5.00	3.2
5–14	346,834	27,461	7.9	6.7
15–34	1,485,416	142,958	9.6	28.7
35–39	1,897,455	198,357	10.5	36.6
60–79	853,872	89,503	10.5	16.5
> = 80	432,601	61,694	14.3	8.4
Total	5,180,350	528,141	10.2	100.00

Source: Robert Koch Institute; laboratory-based surveillance of SARS-CoV-2, 2020-W42-2020-W53, Data as of 05.01. 2021https://ars.rki.de/Docs/SARS_CoV2/Wochenberichte/20210106_wochenbericht.pdf

**Fig. 1:**
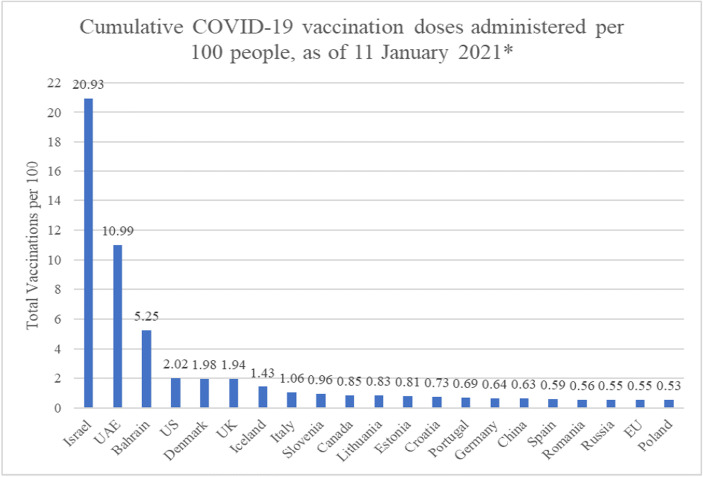
Total Vaccinations Per Hundred, Selected Countries; as of January 11, 2021*. Source: Own representation of data available from Our World in Data (OWID). Note data available as of 11 January, country-level data from January 8–11, except UK (as of Jan. 3), Iceland (as of Dec. 30), Russia (Jan. 2)

Vaccination will become an increasingly important option for fighting the coronavirus pandemic in many countries. Israel, Iceland, the UK, Denmark, Germany, Canada, Slovenia, Portugal, Spain and Luxembourg were the ten leading countries leading in terms of vaccination intensity as of January 11, 2021 (see Fig. 1).

## Corona infection dynamics and a testing & quarantine strategy

The subsequent simple and basic model allows an understanding of the effects of a robust testing strategy. The following reasoning is highly simplified as we intend in the first instance for the reasoning behind the approach to be easily accessible. Thus, the subsequent approach includes very few parameters. However, this is sufficient to make clear the key aspects which have to be considered on the issue of a roll-out of a national testing strategy. Thus far, testing for the novel coronavirus in most OECD countries has been applied in a regional or local context, or even in individual institutions such as care homes and hospitals, while a broader, i.e. national, regular testing strategy could generate broad and significant benefits with respect to both the medical outcome and economic aspects. Epidemics in many countries could be firmly brought under control and the recovery represented by an economic upswing reinforced in the medium term.

Thus, infection dynamics of the novel coronavirus are described using the most basic well-known model of disease dispersion. In this basic model, the number of new infections per day is *ΔI*. As *I* denotes the number of currently infected individuals, the number of new infections is determined by two key factors: (i) the number of contacts per person *c* and (ii) the probability of transmission of the disease *p*. For our basic model therefore, *ΔI = p c I*, the crucial reproduction rate R is

1 $$ R=\Delta \mathrm{I}/\mathrm{I}=\mathrm{p}\ \mathrm{c} $$

**Fig. 2:**
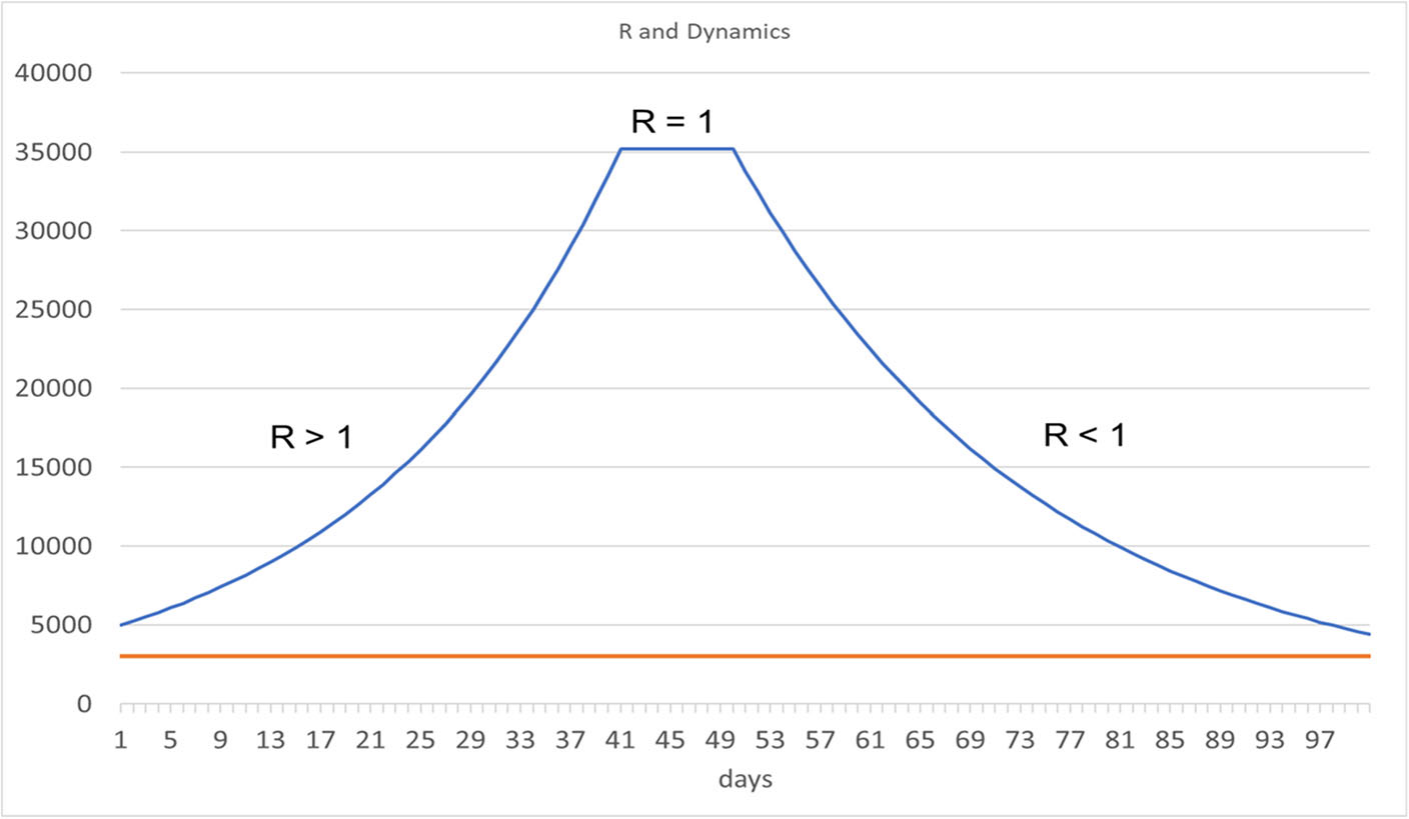
New Infections and Infection Dynamics for Different Values of R*.* Source: Own representation

The R value is critical because it determines if the disease is continuing to spread amongst the population, when R > 1, or if the spread of the disease is starting to reduce, when R < 1. In the example of Fig. 2, the number of new infections doubles every 2 weeks for R > 1. If R = 1, the rate of new infection remains at a constant value. For R < 1 the number of new infections continuously decreases and the pandemic eventually ceases.

Conventional policies intended to control the process of the spread of infections focus on two principle parameters, namely *contacts* and the *probability of transmission*. Social restrictions or even lockdowns are an attempt to minimize contacts, while masks and so-called social distancing rules try to reduce the probability of transmission. While social distancing rules and masks are relatively cheap measures, a lockdown of broad sections of the population is the most expensive measure for an economy. Therefore, it makes sense to look at other measures which provide similar or even better effects at lower cost. Thus, we suggest an intelligent test strategy, T&Q, as being an effective measure at lower cost, both economically and with almost no restrictions in terms of fundamental individual rights (which is another kind of cost factor). The test approach suggested here means randomly testing a chosen fraction of all groups – regardless of whether they have symptoms of a Covid-19 infection or not.

The simplest example for illustrating how a T&Q strategy works can be described by extending the basic Eq. 1. We denote *t* as the share of the population tested. If currently infected persons go on to infect *pc* additional people, and share *t* of the population is tested, the same share *t* of the newly infected people *pc* will also be tested. Thus, this share of just infected people will have a positive test result and can be identified as sources of spread of the disease. If this positive tested share *t* immediately goes into quarantine, it cannot contribute to new infections so that the total number of new infections decreases by this share. The reproduction rate for this simple T&Q strategy can now be described as:

2 $$ \mathrm{R}=\left(1-\mathrm{t}\right)\ \mathrm{p}\ \mathrm{c} $$

This equation indicates that the reproduction rate can be reduced via a T&Q strategy which is here described by the share of people who test positive *t* and are sent into quarantine. Let us provide a numerical example. Assume the probability of transmission is *p* = 4%, and let us assume that the average number of contacts of an infected person during the period that person is infectious is on average *c* = 30 people. Let us further assume that the government introduces a broad testing strategy as suggested herein according to which 20% of population (*t*) is tested, thus also 20% of the newly infected individuals can be quickly identified through a test. Thus, that 20% of the newly infected people are quickly isolated by quarantine measures and cannot contribute to the further spreading of the disease. As the share *t* = 20% of newly infected people is in quarantine, the total is reduced by this *t* = 20% and we obtain a reproduction rate for this example of less than 1:

3 $$ \mathrm{R}=\left(1-0.2\right)\bullet 0.04\bullet 30=0.96. $$

Without the T&Q strategy the reproduction rate is R = 0.04*30 = 1.2, that is R > 1. With a T&Q strategy and a reproduction rate of R < 1, the process of the spread of infections is quickly suppressed as we can see in Fig. 2. As *t* = 20% is the rate of newly infected individuals that is immediately tested, isolated and put into quarantine, this share of newly infected people cannot contribute to a further spread of the disease. The reproduction rate changes from an expansionary process (R = 1.2 > 1) to a process of falling numbers of infected (R = 0.96 < 1). Thus, a broad T&Q strategy is another effective instrument to control the pandemic. The central question is: How expensive is this strategy compared to other control strategies, for example the lockdown, which basically reduces social contact. In order to keep costs low, we need to develop a clever test strategy. This strategy would drive the overall reproduction rate below unity at the lowest possible cost of testing. There are many sophisticated strategies which consider a smart testing strategy, however for the purposes of the present paper we want to go through a relatively parsimonious example which, despite its simplicity, is a clear blueprint for new strategies.

**Fig. 3:**
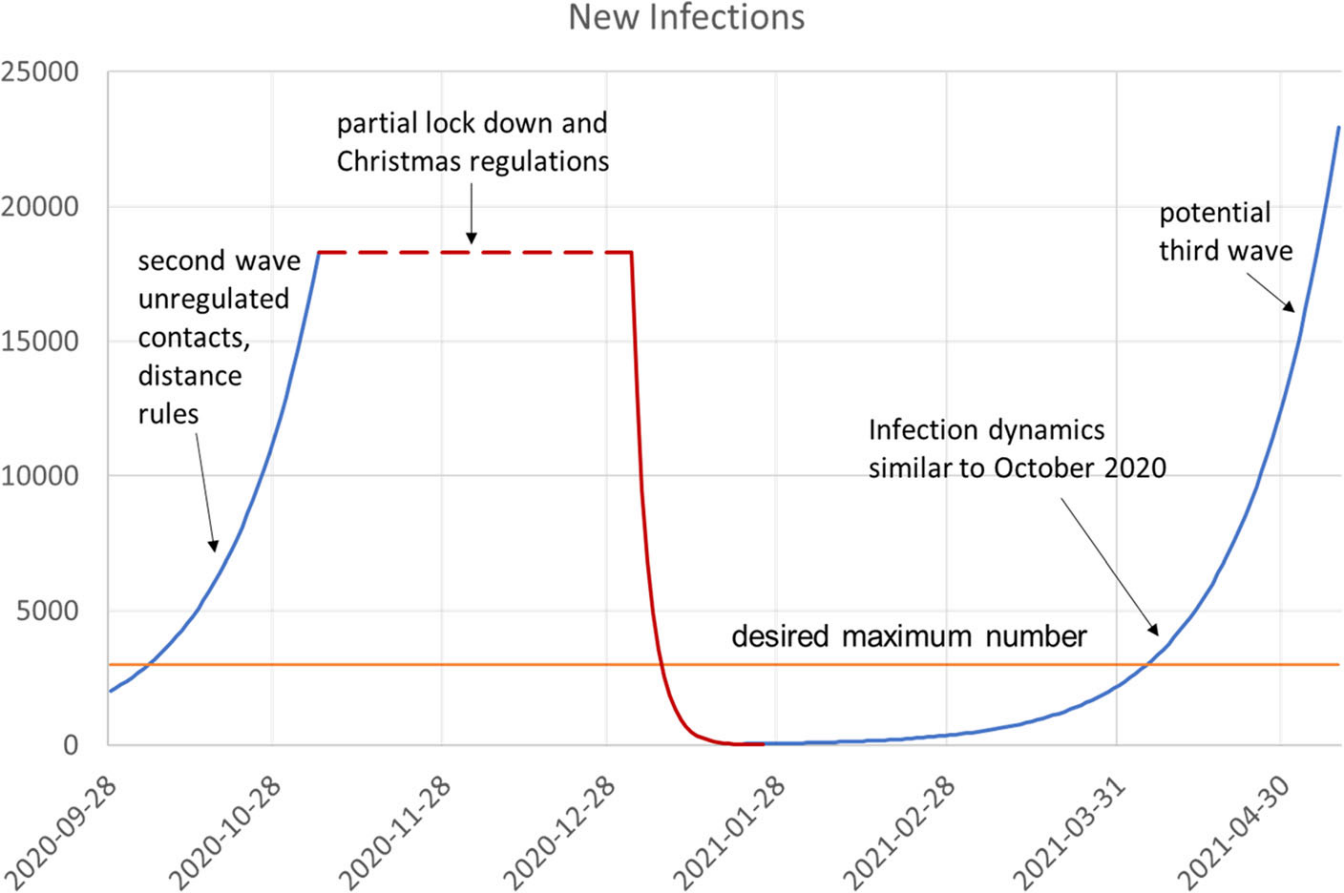
Example of an Infection Scenario. Source: Own representation, Data source for calibration of model: WHO; Johns Hopkins University (2021)

With this simple stylized scenario (with reference to Germany as an example), we want to illustrate (i) how such a T&Q strategy would work, and (ii) how much it would cost. Figure 3 describes a time span of about half a year from October 2020 until May 2021. We take the detected infections as an indicator of infection dynamics in October which close to the real observed numbers in that month. During this first period, numbers grow exponentially until this growth was restricted by the first “partial lockdown” which was implemented in November. This is indicated by the blue line which always describes the unrestricted growth. The dashed red line describes the spread during the “lockdown lite”. In this stylized example, we do not look at the various interaction effects between the full lockdown and the lifting of restrictions over the holiday period or “Christmas deregulations”. We simplify and assume that the effective full lockdown starts at the beginning of January 2021. Interpersonal contacts are drastically cut and at the beginning of February new infections are down to the desired level. However, this is not the end of the story. When the deregulated phase starts, the growth in the number of infections returns. If we simply assume that the dynamics under unregulated conditions is similar to October, we obtain a third wave in April. The blue curve shows this process. Even if a vaccination exists, it will not be quickly available for the majority of the population, and thus it cannot help to solve the problem of a third wave. Further, more aggressive variations and mutations of the virus are likely to exacerbate infection dynamics, such that a faster spread becomes more likely.

Without another instrument to control the pandemic in Germany, this third wave - and the required third lockdown - would lead to the same massive problems as were observed in November and December 2020. The costs are enormous, in terms of lives lost to the disease, restrictions of fundamental individual rights, income, insolvencies and unemployment et cetera. Therefore, we would like to encourage a broad discussion about an alternative policy measure and suggest the T&Q strategy as a bundle of systematic test and quarantine rules.

### T&Q strategy

The basic idea is thus: Rather than isolating everybody in a further lockdown, a systematic testing strategy detects and isolates only those individuals who are already infected or potentially infected with a high chance (as they had close contacts with infected persons). It is a systematic, statistical detecting and protection strategy.

As previously mentioned, we do not intend to provide a sophisticated simulation here, but we can suggest a calculation that illustrates how this strategy would work and argue that it is worth to consider this strategy as an important alternative to the most expensive instrument, namely the lockdown. The purpose is to provide simple calculations to introduce the elements which should play a major role for the development of such a test strategy. For the example above in Eq. 3 we could already demonstrate that a test share of *t* = 20% of the population would suppress the pandemic. While this example is a simple statistical model using overall average numbers, the efficiency of a strategy increases enormously if we look at different groups and their contributions to the spread of disease. Considering specific groups can massively reduce required test capacities and reduce costs. Groups that have more contacts and this a higher likelihood of spreading the disease should be tested more often than others.

In our example, we simply take different age groups and presume that these age groups have different contact frequencies. Table 5 describes the different age groups for Germany, their share of total population and their absolute numbers.

**Table 5:** Population of Germany and breakdown by age group

	Total	Age > 80 yrs	60–79 yrs	30–59 yrs	20–29 yrs	0–19 yrs
Share in %	100.0%	6.0%	21.6%	41.9%	12.0%	18.4%
Million inhabitants	82.5	5.0	17.8	34.6	9.9	15.2
R factor per day^a^	1.06	0.50	0.75	1.23	1.23	1.20

Source: Own representation; data Source for population age groups Statistisches Bundesamt (2021), Data Base Genesis-Online for the year 2016

^a^The R factor per day is based on model assumptions, but reflects the real dynamics in October 2020 in Germany (see also Fig. 3)

Different age groups are characterized by different living conditions which affect both contact frequency and the probability of transmission of the disease. For instance, young people are at school and regularly meet in class or during sport classes or other hobby activities. Their number of contacts during the spreading phase is quite high and at school there is little chance of appropriate and adequate distancing. Thus, a high number of contacts with a high probability of transmission may lead to a relatively high R_i_ factor. The working age group, often families, have contacts at their job and they also frequently meet people to engage in leisure activities, socializing and hobbies. For this group, we also have a relatively high frequency but very likely more distancing etc. As a result, each group has a group specific contribution to the overall spread of the virus and thus the R_i_ factor. With some plausible guesses of *p* and *c*, we estimate group-specific R_i_ factors per day as described by line three in Table 5. The overall R factor per day is calculated as R = 1.06 from the assumed R_i_. However, the overall R factor is consistent with the growth of new infections through the October period of less regulated (no lockdown) spreading.

As a consequence of group specific contributions to the spread of infection, we need a group specific testing strategy. Groups with a large number of contacts and a large probability of transmission must be tested more frequently than groups with relatively few contacts. For instance, school students and teachers have contact with many other students and teachers and thus it is not easy to reduce the probability of transmission due to narrow space and a low level of protection measures. This group is likely to have a high group specific R_i_ and to contribute highly to the overall R. However, if this group is systematically tested with a high frequency, the effective R_i_ of this group can become very low and the overall R is massively reduced.

Moreover, if an individual is tested and proven to be infected we need a strict quarantine policy. For instance, if a student tests positive, the whole school class may be sent into quarantine. That is, people closest to the originally infected person are most likely to be infected as well. Thus, these people are prevented from spreading the disease any further. The public is protected. If this group is large - like in a school setting - the test has a high effectiveness. That is, for each group, the number of systematic contacts must be identified as well as the share of people who do not behave properly – meaning that they do not accept or follow the quarantine rules. To address these arguments, we slightly extend the basic model of Eq. 2 and introduce the parameter *q* for the effectiveness of the quarantine. In addition to all these elements, we also include in this parameter *q* the willingness to be tested and the test reliability (probability of a wrong negative result) such that *q*_*i*_*∙t*_*i*_ is the Testing & Quarantine coverage share

4 $$ {R}_i=\left(1-{\mathrm{q}}_i\bullet {\mathrm{t}}_i\right)\ {p}_i\bullet {c}_i. $$

To determine the overall result of the test strategy and obtain the overall reproduction rate R we only need to weight each specific R_i_ factor with the population shares for each group and add up all weighted R_i_ to the total factor.

5 $$ \mathrm{R}={\sum}_{i=1}^n{w}_i\bullet {R}_i,\kern0.5em \mathrm{with}\ {w}_i\ \mathrm{being}\ \mathrm{the}\ \mathrm{weight}\ \mathrm{of}\ \mathrm{respective}\ \mathrm{population}\ \mathrm{groups}. $$

For our example scenario, Table 6 defines the test strategy. The table describes the frequencies of testing for each age group and the implied coverage ratio as a result of further assumptions about the effectiveness of the quarantine strategy.

**Table 6:** Parameters and results of the suggested T&Q strategy

	Total	Age > 80	60–79	30–59	20–29	0–19
Tests per person and week		0.25	0.25	1	0.5	1
Test reliability		85%	85%	85%	85%	85%
Willingness to be tested in contact groups		65%	65%	65%	65%	65%
Share of contact groups that moves into quarantine		80%	20%	10%	5%	10%
Number of people who are quaran-tined as result of a positive test		8	5	5.5	1.75	3
Test effectiveness (q_*i*_ ∙ t_*i*_)		0.16	0.10	0.28	0.07	0.24
R-tested	0.84	0.34	0.68	0.89	1.14	0.92
Million tests per day	8.63	0.18	0.64	4.94	0.71	2.17

Source: Own representation

**Fig. 4:**
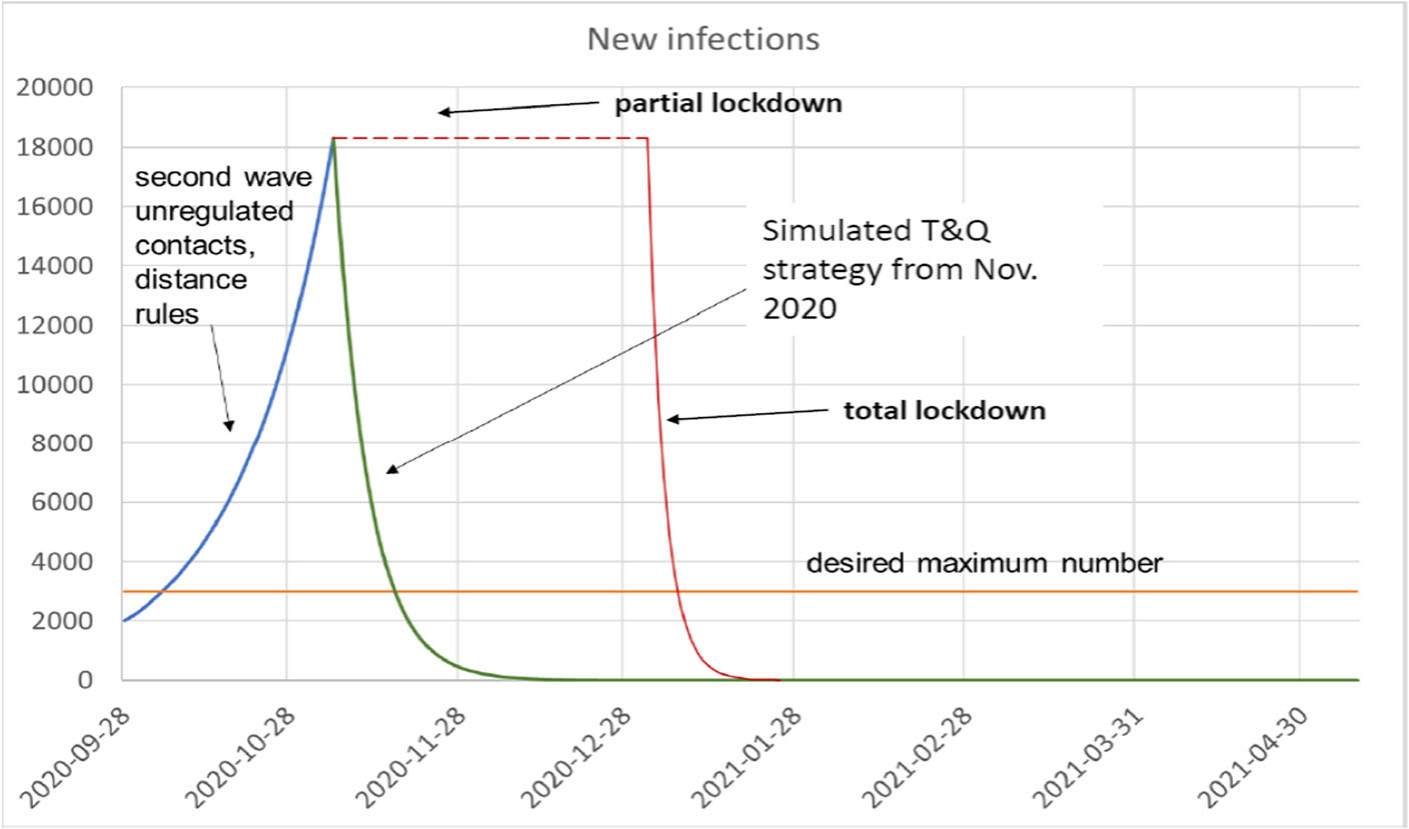
Scenario for a Test Strategy. Source: Own representation

The result for this example of a T&Q strategy is drawn as the green line in Fig. 4. To make an interesting case for comparison, we assume that we had started this test strategy already at the same date in autumn (November) when the partial lockdown started in Germany. According to the graph, this test strategy with a systematic and group-specific testing approach had brought down new infections within a month to a level far below the desired maximum level which is indicated by the orange line. Thus, this example shows that a systematic test strategy can be effective. Even more, this simple strategy suggested in Table 6 allows to reduce the required tests to a number of less than nine million tests a day. With a continuous testing, the number of new infections is continuously kept at a very low level. The third wave will not appear.

#### Cost issues

**Fig. 5:**
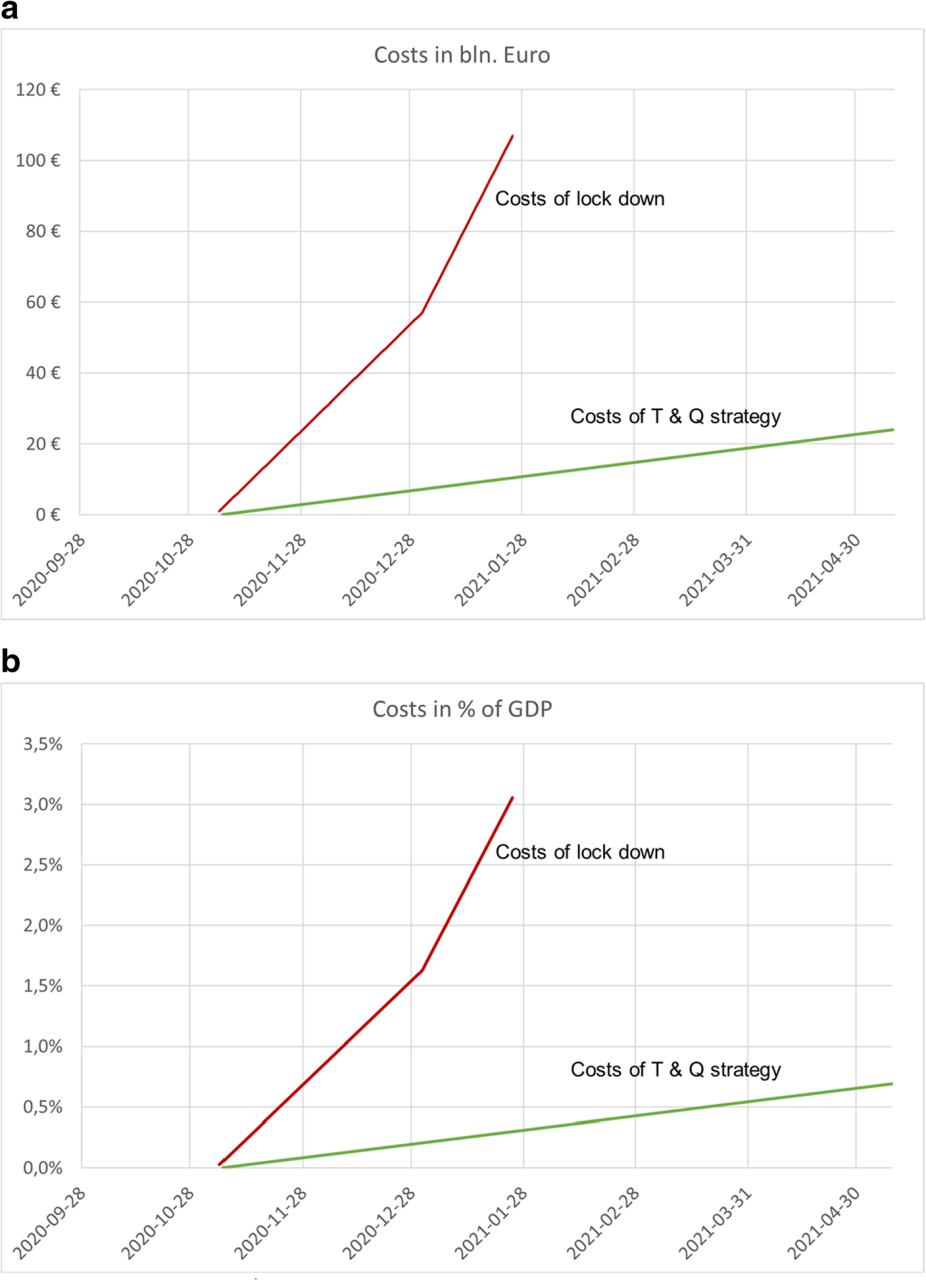
**a** Costs of Different Instruments, Lockdown versus Test Strategy in Billions of Euro. **b** Costs of Different Instruments, Lockdown versus Test Strategy as Percentage of GDP. Source: Own representation

However, an argument against the systematic T&Q strategy is the high cost of testing. Thus, let us now consider the cost of our proposed approach. Estimated lockdown costs are collected in Table 2. The most often communicated figure for the economic loss suffered by Germany is an estimate −5.5% of GDP for 2020 by Germany’s Council of Economic Advisors (Sachverständigenrat (2020), p.40). However, this is the total loss throughout the whole year. If we look more directly at the loss of GDP for the second quarter of 2020 compared to the second quarter of 2019, we can estimate a loss of 10% which is a total amount of around €86 billion.^1^ If we relate this loss to the 30 days of the lockdown in that quarter, we obtain a loss of €2.8 billion per day. However, in our calculation, we are more conservative and estimate €1 billion per day for the partial lockdown in autumn 2020 and €2 billion for the full lockdown. The red lines show the results in Fig. 5, both in absolute terms and as a percentage of GDP.

Next, we need to calculate the cost for the test strategy. Fortunately, since autumn 2020 rapid tests are available at a cost of less than €5. However, a network of dedicated infrastructure is also needed to perform and evaluate the testing. Let us assume that infrastructure cost per test is 3 times the cost of the test itself (which is probably much too high), such that a test on average costs €15. How are total costs developing if we follow our test strategy? Figure 5 gives the answer, both in absolute values (Fig. 5a) and as percentage of GDP (Fig. 5b). The green line gives the costs generated by the costs of testing. It is slowly but continuously increasing the longer the strategy is applied. If the test volume of nine million tests a day would be kept at that level for a whole year total cost would not considerably exceed 2 % of GDP - if at all due to scale economies. Thus, the costs of the T&Q-strategy are much less than the costs of lockdowns. However, apart from costs, the most important benefit can be clearly identified. Most private and business activities can continue in a relatively normal fashion as long as simple distancing rules are still enforced (we have assumed no change in social distance rules compared to the summer of 2020).

Furthermore, it should be noted that progress with vaccinations effectively reduces parameter *p* in Eq. 1, but it might raise for some parameter *c* as people yet to receive a vaccination could become less concerned about the risk of infection. In the medium term, however, the term *pc* can be expected to reduce along with progress in terms of the vaccination program for the general population and this, in turn, will contribute to a reduced need for testing and therefore also help to bring down the overall cost of the T&Q strategy. The cost of the T&Q strategy over time will also come down once the level of infections has strongly reduced which, in turn, with a rather modest testing coverage – and hence a reduced cost relative to GDP – will allow keeping R below unity so that a general spreading of the infection can be safely avoided. If there are mutations of the novel coronavirus which make it more infectious, the parameter *p* is raised which would mean that a higher testing parameter *t* should be applied. One should also note that the parameters used in our scenario table could differ for various OECD countries and other countries in the world economy. However, the powerful implications of our basic approach will remain valid. The approach suggested herein is thus a robust approach for various settings and certainly should be very a useful blueprint to policymakers as long as progress with vaccination programs worldwide has not achieved herd immunity in all countries.

## Policy conclusions

This analysis shows that a careful Testing & Quarantine strategy can help to avoid the need for further broad lockdowns/shutdowns altogether – hot-spot regions could still face localized lockdowns in some countries, but national lockdowns/shutdowns can clearly be avoided if policymakers would follow the suggested approach developed here. Producing an adequate number of testing units is a key requirement for establishing a broad national testing strategy. Policymakers could have offered large contracts – often with certain options (as a means to have some flexibility for coping with the changing situation) - and adequate incentives for a comprehensive testing infrastructure in OECD countries as early as spring 2020, but they apparently did not; with the exception of schools, hospitals and care homes. As regards workers in nursing and care homes, typically only those with symptoms were tested leaving those with asymptomatic infections without any “signaling test”, so that some super-spreaders have emerged even from the crucial care sector (this has happened, for example, in Tübingen, Germany, prior to a new general testing strategy for care homes in summer 2020). By contrast, the approach presented herein emphasizes the advantage of random testing and relies on the frequent testing both people with symptoms and without symptoms – and it is well known that many people infected with the coronavirus indeed exhibit no symptoms.

This policy-oriented paper is complementary to the IMF paper of Cherif and Hasanov (2020), but our approach is in clear contrast to the dominant approach in the EU as represented by the European Center for Disease Prevention and Control (ECDC). The ECDC wrote in September 2020 that people with symptoms should “be tested as soon as possible after symptom onset…Healthcare and social care settings require intensive testing when there are documented community transmissions. Periodic and comprehensive testing of all staff and residents/patients is recommended to prevent nosocomial transmission.” (ECDC 2020, p. 1). This is an analytically flawed recommendation since it overlooks the generally crucial option of including in a broad testing approach those infected people without exhibiting symptoms who could be included at least in a random sampling approach in a consistent way as proposed here; the approach presented herein, does not, of course, exclude full testing in care homes. Limited testing restricted only to those people with symptoms was adequate in the very early stage of the pandemic when test lab capabilities and test sets were extremely scarce. It is disappointing that the testing strategies suggested by the ECDC on September 15, 2020, had not been modified compared to the early stages of the coronavirus pandemic in early 2020.

It is obvious that some quarantined people will not accept the quarantine and try to bypass it so that it would be adequate to impose significant fines for violations of the quarantine. A national testing approach requires the building of a costly testing infrastructure which, however, could in many cases be based on functional links to existing institutions: say, large firms, schools, universities or public administration entities. Such an approach reduces the costs of testing in a useful way. Every city must have a quarantine testing station and the availability of special quarantine hotels in which special groups of people with a positive test result could be accommodated (e.g., travelers, families in very small apartments and so on) should also be considered as part of the local toolbox.

Raising the production of tests should not be a major obstacle in OECD countries, newly industrializing countries or developing countries. The costs of now fairly established standard rapid corona tests have come down considerably in 2020. There are also quick mobile Corona PCR tests available (e.g., from the Spindiag GmbH, a German start-up whose equipment delivers test results within 40 min; the equipment has already been used in a major clinic in Stuttgart; in the Appendix, we also refer to figures from long-term care facilities in Maryland which show that not testing those without symptoms creates serious problems and inefficiencies). The argument that these rapid tests are not reliable enough is also not problematic for the overall effectiveness of the proposed strategy. Since it is a statistical testing strategy, low test reliability can be compensated for with a higher test rate. This has also been taken into account in our simulation.

One can easily anticipate that many politicians will be hesitant to support a Testing & Quarantine strategy since a) this is a new proposal within the broader Corona perspective and b) since the budget-related expenditures of the T&Q approach in the first half of the year when implemented are rather high. However, there are clear arguments that the cost/benefit analysis is clearly in favor of the T&Q strategy (plus a complementary vaccination program). A continuation of the current cyclical lockdown strategy in most OECD countries - compared to the broad T&Q approach presented here - implies an immediate loss in aggregate national income which is at least three times as high. Moreover, the T&Q approach avoids restrictions on fundamental rights, long-term negative effects on educational opportunities and the threatened of wave of insolvencies which would follow prolonged lockdowns. From this perspective, it is important to start a broader public debate which really makes clear how important T&Q (combined with progress in terms of vaccinations) is for the population and the economy. This new strategy can save many lives and avoid millions of infections while greatly helping to reinforce the economic upswing. The approach suggested here can be applied in OECD countries, as well as in all other countries of the world economy. Give then €750 billion EU Corona recovery budget, it should not be a problem for Germany and other EU countries to quickly implement the suggested Testing & Quarantine strategy. An improved coronavirus warning app should also be part of the broader policy modernization package to fight the pandemic. Young adults in particular should be offered a special incentive to actively use the corona warning app. Fighting the epidemic through a broad Testing & Quarantine strategy is the mildest form of policy intervention if one takes into account the medical advantages, the economic costs and benefits plus the maintaining of liberty for individuals. There is definitely no time to waste in altering the course of the epidemic policy in OECD countries and so many other countries.

Following an adequate Testing & Quarantine strategy means that lockdowns can be avoided while still controlling the coronavirus epidemic. The proposals presented herein should, of course, not be interpreted as meaning that authorities and researchers worldwide should relax efforts to quickly produce and distribute more vaccines worldwide.

The political economy of epidemic policy reform is complex and once an international institution such as the European Commission - or national governments in the EU member states - have picked up the quasi-official line of a scientific organization such as the European Centre for Disease Prevention and Control, it seems difficult to correct the testing approach suggested by such an institution. However, taking the new insights from Cherif and Hasanov (2020) and from the present study about the functioning, effectiveness and costs of a T&Q strategy, into account, an urgent interdisciplinary scientific discourse and a new political debate become necessary. Within the framework of this debate, the proposed T&Q strategy can be evaluated and further developed, and this debate should modify as well as add to the policies proposed thus far in an effective way. This is what we aim to initiate with this policy-oriented paper.

## Figures and Tables

**Fig. 6:**
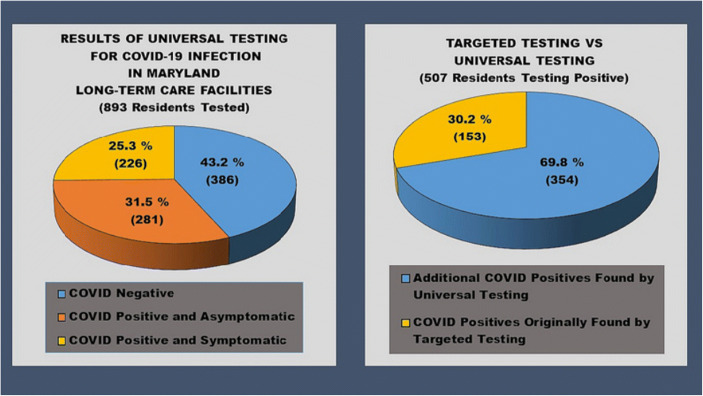
COVID-19 Story Tip: Universal Testing May Help Reduce COVID-19 Infections, Deaths in Long-Term Care Facilities 07/14/2020*.* Source: Johns Hopkins University News Release (2020) https://www.hopkinsmedicine.org/news/newsroom/news-releases/covid-19-story-tip-universal-testing-may-help-reduce-covid-19-infections-deaths-in-long-term-care-facilities

## Appendix

**Table 7:** Total tests for COVID-19 per 1000 people in 35 OECD countries

Total_Ctests_per_thousand	31.03	30.04	31.05	30.06	31.07	31.08	30.09	31.10	30.11	31.12*
Luxembourg	26.24	69.74	123.88	306.23	779.47	1043.30	1354.45	1751.36	2222.25	2661.80
Denmark	5.17	43.45	108.78	183.83	266.07	428.62	670.97	916.44	1281.22	1813.22
Israel	8.99	44.03	66.44	115.69	205.06	285.37	432.20	561.49	696.24	933.14
UK	2.29	13.59	51.72	92.40	150.78	228.42	330.97	464.67	603.80	766.85
US	4.55	21.62	57.85	112.72	196.29	273.35	354.53	461.07	600.24	749.20
Iceland	57.07	143.87	179.04	192.92	210.78	263.97	380.61	545.11	626.99	706.29
Lithuania	3.27	45.12	107.01	150.46	177.02	216.44	264.38	347.53	465.63	602.66
Belgium	5.97	35.14	77.71	109.95	147.45	199.45	284.43	433.13	511.83	600.79
Portugal	7.85	42.07	83.94	118.99	161.68	203.61	260.14	342.21	453.17	522.07
Norway	15.29	30.05	46.39	65.84	90.22	156.27	235.85	315.87	422.98	520.06
Ireland	6.24	34.36	69.79	87.04	128.20	170.12	239.21	331.08	397.89	480.94
Estonia	11.15	44.14	71.74	93.79	105.35	133.47	198.40	257.38	364.30	480.09
Spain		28.90								467.55
Latvia	7.85	30.69	57.83	79.97	105.24	133.45	169.66	240.83	331.11	464.96
Finland	4.25	18.99	35.17	46.40	69.14	132.30	203.90	281.69	360.81	450.10
Australia		22.10	57.04	96.31		243.71	299.51		392.01	441.60
Italy	8.39	32.74	64.15	89.15	112.69	142.98	187.46	261.07	362.96	439.92
Austria		28.47	49.80	68.04	98.87	131.10	179.65	247.91	344.01	426.02
Switzerland	16.02	31.87	46.18	68.97	92.32	118.83	159.91	233.33	309.48	381.49
Canada	6.39	21.37	44.14	73.40	107.32	145.84	194.90	251.76	304.05	372.04
Germany			52.10							372.04
Czechia	5.23	23.71	41.43	51.93	65.35	85.43	129.95	218.17	288.44	356.08
Slovenia	10.06	24.68	36.58	47.49	61.60	74.99	107.77	173.88	249.01	345.63
Chile	1.44	9.05	30.07	57.64	84.53	126.83	173.38	224.98	278.58	337.07
Netherlands			21.19							298.63
N. Zealand	5.20	29.31	57.32	82.21	95.93	156.84	197.83	228.33	264.51	291.54
Turkey	1.10	12.26	24.18	40.10	56.92	84.64	123.08	166.02	220.45	290.55
Greece	1.61	7.21	17.32	30.01		91.08	125.45	170.52	227.72	268.93
Slovakia	1.67	16.68	31.66	38.80	48.44	61.85	85.54	145.02	198.30	264.76
Hungary	1.44	7.53	19.23	28.44	35.09	44.30	74.06	110.24	170.57	230.53
Poland		8.11	22.10	35.84	51.04	68.69	84.49	119.87	158.99	183.41
Colombia				14.89	30.80	51.41	65.52	82.22	99.48	119.24
S. Korea	7.68		17.31	24.44	30.14	36.67	44.91	50.69	58.47	78.87
Japan	0.25	1.16	2.13	3.09	5.57	10.45	14.94	19.29	25.40	35.48
Mexico	0.23	0.84	2.32	4.70	7.85	10.70	13.47	16.63	20.09	25.33

Source: Own representation of data available from Our World in Data (OWID) https://github.com/owid/covid-19-data/blob/master/public/data/owid-covid-codebook.csv

Figures are those recorded on the last day of every month starting from March 2020 (or latest available date – the cut-off date varied across countries in December)

**Table 8:** 7-day incidence of Covid-19 Infections (by calendar week (C.W.) and age group), Germany 2020

C.W.	Total	90+	85–89	80–84	75–79	70–74	65–69	60–64	55–59	50–54	45–49	40–44	35–39	30–34	25–29	20–24	15–19	10–14	5–9	0–4
10	1.07	0.12	0.38	0.33	0.59	0.3	0.51	1.12	1.1	1.98	1.92	1.75	1.23	1.26	1.48	1.52	1.07	0.57	0.27	0.2
11	7.74	1.46	2.43	2.49	2.89	3.95	4.88	7.63	11.15	14.6	14.13	11.61	10.32	9.72	10.8	7.01	4.59	2.78	1.53	0.91
12	26.97	12.03	13.94	14.21	14.73	16.93	18.32	28.29	38.58	46.11	39.75	36.73	33.18	37.32	39.47	31.4	13.73	6.83	4.53	3.61
13	40.92	51.39	47.19	39.01	36.32	36.09	33.96	44.69	53.37	59.63	55.43	47.46	45.13	48.02	53.09	49.34	23.86	10.24	6.68	6.46
14	43.4	142.4	92.4	59.68	46.07	38.54	34.82	49.29	54.44	55.17	51.58	47.04	41.71	44.02	48.97	48.72	27.42	11.59	7.83	6.92
15	32.69	151.39	90.74	52.21	33.87	28.61	22.97	31.85	38.74	38.97	36.41	34.1	31.25	31.86	38.13	37.58	21.78	8.16	6.33	5.93
16	20.88	102.91	59.21	35.91	22	17.2	13.62	19.23	23.27	23.2	22.26	20.84	18.59	21.87	25.56	26.73	14.5	6.37	4.37	4.06
17	14.89	70.71	43.74	24.98	15.17	11.54	10.41	11.93	15.73	14.75	14.94	14.24	14.43	16.54	19.63	20.9	10.21	5.08	4.11	4.64
18	8.95	34.51	20.97	13.66	9.05	7.54	5.47	7.19	8.91	8.99	8.87	9.27	8.79	10.24	13.42	12.91	6.68	3.67	3.19	3.33
19	7.49	25.27	16.82	9.08	7.07	5.63	4.28	5.84	7.55	7.75	8.46	8.25	7.94	8.9	10.21	11.02	6.55	3.38	2.82	3.46
20	5.69	17.13	11.51	7.47	4.46	3.48	2.96	4.23	5.28	5.53	6.28	7.21	6.64	7.17	7.65	8.98	4.59	3.16	2.98	2.9
21	4.35	12.51	8.12	4.61	2.68	2.53	2.55	3.1	3.93	3.73	4.82	4.91	5.22	5.88	6.9	6.81	3.91	2.92	2.17	3.08
22	3.86	8.75	5.5	3.43	2.12	2.18	2.22	2.99	3.52	3.56	4.04	4.34	4.56	4.88	6.25	6.27	4.11	3.21	3.03	2.22
23	2.84	5.1	3.26	2.31	1.5	1.28	1.23	1.91	2.09	2.24	3.09	3.56	2.95	3.75	4.39	4.97	4.11	2.78	2.68	2.73
24	2.82	5.83	3.26	1.91	1.14	1.09	1.05	1.61	2	2.19	2.73	3.22	3.69	4.06	4.79	5.38	2.82	3.21	3.3	2.8
25	4.92	4.5	2.88	1.88	1.68	1.58	1.44	2.14	2.73	4.68	7.11	9.15	7.09	7.33	7.72	8.12	6.14	5.19	4.72	4.04
26	3.86	3.16	2.17	1.55	1.47	1.09	1.34	1.91	3.01	3.96	5.25	5.54	5.69	6.02	6.4	5.56	4.29	3.94	3.73	3.43
27	3.24	2.92	1.79	1.46	1.03	1.03	1.28	1.95	2.32	2.77	4.19	4.36	4.27	4.71	6.23	5.6	3.53	2.81	3.33	3.56
28	2.92	1.82	1.28	1.09	1.03	1.52	1.28	1.65	1.72	2.56	3.43	3.87	3.88	4.73	4.47	5.14	3.76	2.78	3.09	3.69
29	3.64	3.28	1.53	1.18	1.14	1.63	1.46	1.95	2.63	3.11	4.4	5.15	4.75	5.4	6.09	6.55	4.85	4.05	3.7	3.53
30	4.74	2.19	2.3	1.52	1.47	1.69	1.75	2.71	3.59	4.38	6.35	6.56	6.83	6.97	8.08	8.18	6.3	5.16	4.05	4.06
31	5.8	4.74	2.62	2.7	1.83	2.45	1.75	2.71	4.25	5.23	7.32	8.72	7.24	7.72	10.03	11.31	9.24	6.78	5.31	3.81
32	7.29	3.4	2.56	2.09	1.44	2.48	2.49	3.61	4.57	6.21	8.65	9.94	9.72	9.37	12	16.19	13.78	9.83	7.65	4.8
33	9.57	3.04	3.58	2.46	1.83	2.15	2.26	3.42	4.84	7.06	10.88	13.04	12.19	12.74	16.51	25.34	18.84	14.72	10.9	6.97
34	11.53	2.19	1.47	1.94	1.73	2.61	2.47	4.2	6.38	8.32	13.1	14.59	14.39	17.21	23.49	33.57	22.24	15.53	10.2	7.17
35	10.6	3.64	2.05	1.52	1.78	2.53	2.7	4.3	5.24	8.03	11.49	13.42	13.52	15.52	24.08	28.8	21.12	13.04	9.44	5.63
36	10.36	2.92	1.6	1.85	1.93	2.94	2.61	4.36	5.79	8.27	11.61	13.2	12.23	17.07	22.8	27.08	19.5	11.61	8.53	5.81
37	11.74	5.35	5.37	3.98	3.74	4.14	4.61	5.98	8.21	9.97	13.2	13.99	13.16	17.01	24.3	27.97	21.1	13.45	8.67	5.81
38	14.76	8.99	6.59	5.92	4.64	7.05	5.95	8.73	11.3	13.37	16.59	17.13	17.02	21.91	29.19	32.9	26.25	13.93	9.5	7.32
39	15.7	11.42	7.8	6.41	5.96	7.24	6.26	9.54	12.82	14.83	17.03	17.52	18.98	23.71	28.28	34.35	26.53	15.83	10.4	7.65
40	19.14	14.46	10.42	9.59	7.89	9.53	9.3	12.02	16.42	17.93	22.7	21.25	21.7	27.71	34.37	37.6	33.66	19.15	11.5	8.18
41	31.45	22.6	19.5	14.3	13.03	16.09	14.12	22.49	27.68	33.75	38.61	38.07	34.52	43.02	53.01	60.54	52.62	30.76	16.8	13.23
42	50.58	52.37	36.32	25.95	23.37	25.37	26.45	37.52	44.5	53.22	59.17	59.24	58.74	70.25	88.21	94.91	75.24	44.61	26.1	22.37
43	89.97	107.53	72.64	55.67	41.89	48.8	46.68	70.05	85.43	95.51	110.08	107.94	101.85	122	144.03	163.74	123.1	74.02	49.2	36.96
44	133.66	177.88	113.82	86.3	68.26	78.44	73.04	106	132.8	145.51	158.87	160.07	152.74	174.1	203.47	226.3	182.4	110.32	74.9	56.72
45	151.23	208.49	145.93	104.2	78.6	86.36	87.24	121.2	150.6	163.05	179.51	180.34	169.21	190.5	219.57	254.84	213.2	128.76	90.1	61.27
46	153.72	280.79	180.78	115.2	84.77	89.49	82.61	122.1	152.8	163.71	175.9	182.68	173.47	190.5	210.56	239.41	220.5	144.26	97.1	61.29
47	154.55	372.28	215.24	132.3	87.35	91.29	85.35	129.3	152	161.29	183.13	188.1	173.73	183.8	195.71	221.81	208	142.94	105	60.23
48	148.32	425.74	261.54	145.3	90.08	86.99	84.87	123.6	146.1	153.81	171.35	178.77	164.64	170.1	176.85	193.99	189.2	139.54	102	59.07
49	154.27	503.13	292.94	164.7	102.03	94.53	84.93	135.4	153.3	156.97	174.75	179.49	172.39	171.8	179.57	199.2	195.7	138.84	98.4	58.59
50	187.52	655.25	377.28	213.4	128.24	118.83	105.8	166.2	192.6	196.57	209.51	220.82	206.68	208.8	211.92	233.38	223.6	152.42	114	68.01
51	208.59	709.8	436.82	247.8	152.28	139.93	128.77	193	220.3	220.57	233.57	241.86	228.75	234.7	237.19	261.09	213.7	140.89	114	78.91
52	163.5	619.65	362.83	219.2	127.44	117.53	111.81	157.4	177.9	173.76	179.39	188.94	175.36	179.2	182.5	193.17	144.9	89.12	79.5	53.97

Source: Own representation of data available from the Robert Koch Institute

“A 2020 study from Johns Hopkins Research shows that for long-term care facilities, it is critical to test all residents for COVID-19 infection rather than target only those who show symptoms. Broads, or universal testing, can identify both symptomatic and asymptomatic cases in a population, both of which are threats for spreading the disease. Thus, a program of broad, randomized testing would be more successful in identifying carriers of the infection, especially asymptomatic patients who may be spreading the infection”; see also Bigelow et al. (2020).

## Footnotes

1 Based on numbers of the Statistische Bundesamt 2020, Destatis, Business Cycle Indicators, Quaterly GDP.
